# A rare case of concomitant huge exophytic gastrointestinal stromal tumor of the stomach and Kasabach-Merritt phenomenon

**DOI:** 10.1186/1477-7819-5-59

**Published:** 2007-06-01

**Authors:** Taiji Watanabe, Kohei Segami, Takahiro Sasaki, Hatsuya Kawashima, Takeharu Enomoto, Yuji Jinnouchi, Satoshi Koizumi, Naotaka Tobe, Joh Sakurai, Tsukasa Shimamura, Tadashi Suda, Takeshi Asakura, Hiroshi Nakano, Tanaka Ichiroh, Takehito Otsubo

**Affiliations:** 1 Department of Gastoenterological and General Surgery, St. Marianna University School of Medicine 2-16-1, Sugao Miyamae, Kawasaki, Kanagawa, Japan

## Abstract

**Background:**

We report an extremely rare case of concomitant huge exophytic GIST of the stomach and Kasabach-Merritt phenomenon (KMP).

**Case presentation:**

The patient was a 67-year-old man experiencing abdominal distension since September 2006. A physical examination revealed a 25 × 30 cm hard mass that was palpable in the middle and lower left abdomen minimal intrinsic mobility and massive ascites. Since the admitted patient was diagnosed with DIC, surgery could not be performed. The patient received a platelet transfusion and the DIC was treated. Due to this treatment, the platelet count recovered to 7.0 × 10^4^; tumor resection was performed at 16 days after admission. Laparotomy revealed a huge extraluminal tumor arising from the greater curvature of the stomach that measured 25 × 30 cm and had not ruptured into the peritoneal cavity or infiltrated other organs. Partial gastric resection was performed. The resected mass measured 25 × 25 × 20 cm. In cross section, the tumor appeared hard and homogenous with a small polycystic area. Histopathology of the resected specimen showed large spindle cell GIST with >5/50 HPF (high-power field) mitotic activity. The postoperative course was uneventful, and the coagulopathy improved rapidly.

**Conclusion:**

Since the characteristic of tumor in this case was hypervascularity with bleeding and necrotic lesions, coagulopathy was thought to be caused by the trapping of platelets within a large vasculized tumor mass.

## Background

Gastrointestinal stromal tumors (GISTs) are mesenchymal tumors of the digestive tract with various clinical and biological characteristics. The expression of c-kit distinguishes GISTs from true leiomyomas, leiomyosarcomas, and other mesenchymal tumors of the GI tract [1-3]. The stomach (60%–70%) and small intestine (20%–30%) are the most common sites for GISTs [1,2].

An association between a vascular lesion and life-threatening coagulopathy is termed as the Kasabach-Merritt phenomenon (KMP). It includes thrombocytopenia, microangiopathic hemolytic anemia, and disseminated intravascular coagulopaty (DIC). Generally, KMP is the most characteristic symptom of giant hemagiomas [4-7]. To our knowledge, no case of concomitant GIST and KMP has been reported thus far. Here, we report an extremely rare case of concomitant huge exophytic GIST of the stomach and KMP.

## Case presentation

The patient was a 67-year-old man experiencing abdominal distension since September 2006. In October 2006, the abdominal fullness became progressive, and he had hard general fatigue. There was no history of vomiting, fever, or gastrointestinal bleeding. Renal failure occurred due to diabetes mellitus (DM). On admission in November 2006, a physical examination revealed a 25 × 30 cm hard mass that was palpable in the middle and lower left abdomen minimal intrinsic mobility and massive ascites.

**Figure 1:**
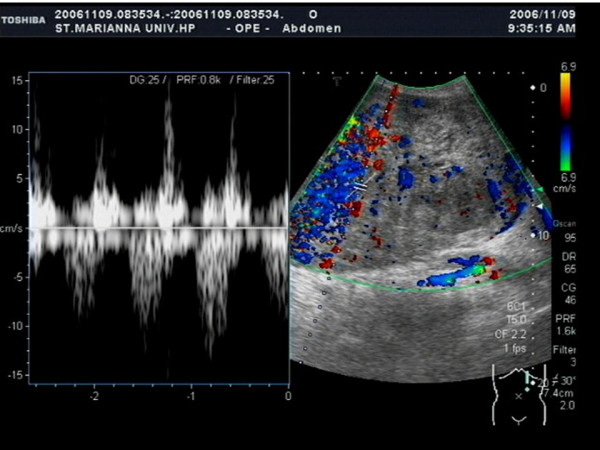
Doppler ultrasonography shows hypervascular area in the tumor.

**Figure 2:**
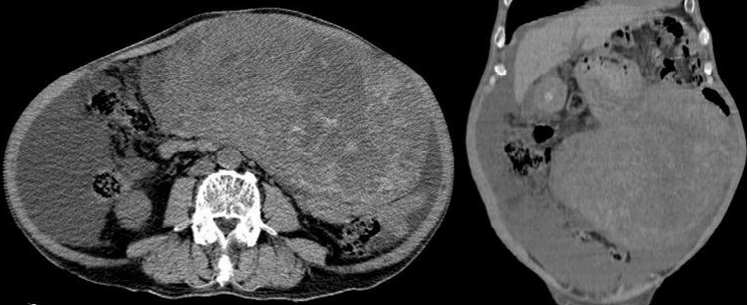
Abdominal computed-tomography shows a huge heterogeneous mass with a thick and irregular wall sized 20 × 25 cm extending from the greater curvature of the middle of the stomach.

**Figure 3:**
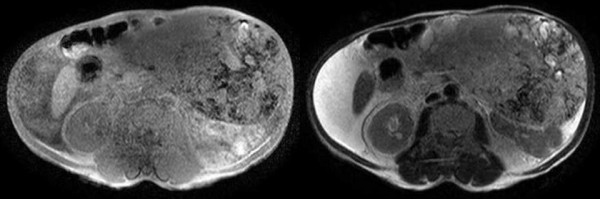
Abdominal MRI shows huge inhomogeneous mass lesion can be identified in the right side of the intra-abdomen.

An upper gastrointestinal barium study and gastric endoscopic examination indicated no anomalies. Tumor findings of Doppler ultrasonography showed a hypoechoic lesion with a hypervascular area (Figure 1). An abdominal computed tomography (CT) scan showed a huge heterogeneous mass sized 20 × 25 cm extending from the greater curvature of the middle body of the stomach (Figure 2). Abdominal magnetic resonance imaging (MRI) was performed, and almost the entire abdominal cavity was visualized in the coronal view. The irregular wall of the lesion exhibited a low intensity signal on the T1-weihted image and a high-intensity signal on the T2-weighted image (Figure 3).

**Figure 4:**
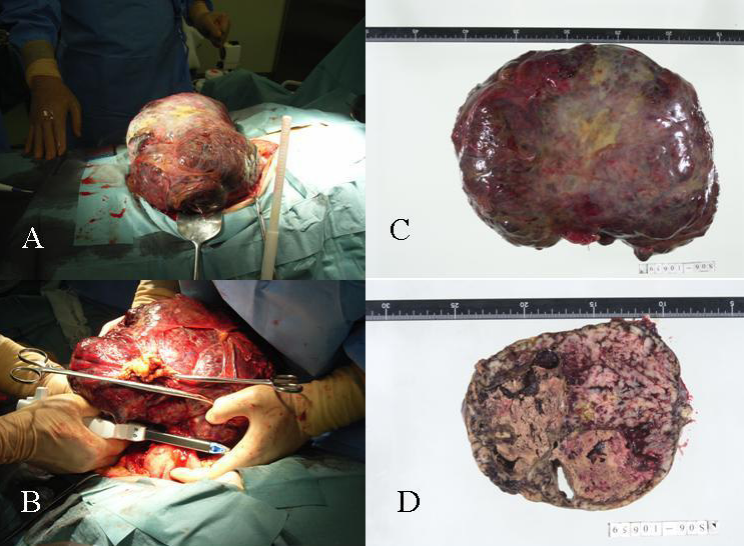
A and B) huge solid tumor arising from the greater curvature of the stomach that measured 25 × 25 cm and had not ruptured into the peritoneal cavity or infiltrated other organs. An attaching pedicle approximately 3 cm in breadth was observed in the greater curvature of middle body of the stomach. Partial gastric resection was performed. C and D) The resected mass measured 25 × 25 × 20 cm. In cross section, the tumor appeared hard and homogenous with a small polycystic area and calcification.

Routine biochemical investigation revealed hypoalbuminemia, renal dysfunction, and hyperglycemia. Since the admitted patient was diagnosed with DIC (platelet count: 2000 mm^3^, FDP: 135.4 μg/ml, PT: 1.20, SIRS score: over 3 heads) surgery could not be performed. The patient received a platelet transfusion of 20 units each day 6 times, and the DIC was treated with nafamostat mesilate and fresh-frozen plasma. Due to this treatment, the platelet count recovered to 7.0 × 10^4^; tumor resection was performed at 16 days after admission. Laparotomy revealed a huge extraluminal tumor arising from the greater curvature of the stomach that measured 25 × 30 cm and had not ruptured into the peritoneal cavity or infiltrated other organs (Figure 4A). An attaching pedicle approximately 3 cm in breadth was observed in the greater curvature of middle body of the stomach. Partial gastric resection was performed (Figure 4A).

**Figure 5:**
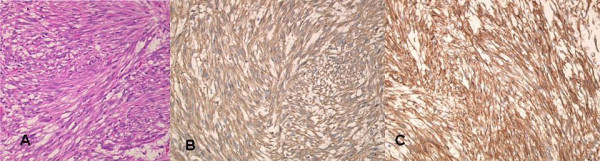
Photomicrograph showing A). Microscopically, the tumor was characterized by fascicular and interlacing proliferation of the spindle-shaped cells.(Hematoxylin and Eosin × 200) B). Histopathology slide after Immunostaining for CD34: Tumor cell show positivity after CD34 staining. C) Histopathology slide after Immunostaining for CD117: Tumor cell show positivity after CD117 staining.

The resected mass measured 25 × 25 × 20 cm. In cross section, the tumor appeared hard and homogenous with a small polycystic area (Figure 4B). Histopathology of the resected specimen showed large spindle cell GIST with >5/50 HPF (high-power field) mitotic activity (Figure 5A). No evidence of infiltration was observed in the resected margins of the stomach wall. Immunohistochemical staining was strongly positive for CD34 and CD117 (Figure 5B and 5C), and negative for α-SMA, S-100 and Desmin.

The postoperative course was uneventful, and the coagulopathy improved rapidly. The patient was carefully followed up regularly. Imatinib mesylate (Gleevec™/Novartis Pharma AG, Basel, Switzerland) was administered orally 300 mg per day because the patient displayed renal dysfunction (serum creatinine: 3.98, blood urea nitrogen (BUN): 41.5)

## Discussion

The definition of a GIST has been changing with immunohistochemical and molecular technical advances [8]. It is widely accepted that a GIST is a mesenchymal tumor that expresses the c-kit oncoprotein or has a mutation in either the c-kit or the platelet-derived growth factor receptor-alfa (PDGFRA) gene [9]. Several studies have revealed various types of c-kit mutations, including mutations in the juxtamembrane (exon 11), extracellular (exon 9), and tyrosine kinase (exon 13 and 17) domains [9].

Fletcher *et al*., reported a classification for malignancies that is based on tumor size and the number of mitotic divisions [10]. They classified gastric GISTs into the following four groups: very low risk (<2 cm in size and <mitoses5/50 HPF), low risk (2~5 cm and < mitoses 5/50 HPF), intermediate risk (<5 cm and < mitoses/50 HPFs or 5–10 cm and <5/50 HPF), high risk (>5 cm and >5/50 HPF or >10 cm and any mitoic rate or any size and >10/50 HPF). According to this classification, our case was classified as high risk. Moreover, Carrillo *et al*., reported that a high MIB-1 index (>22%, in the most active area) was the most powerful predictor of poor survival [11]. The highest MIB-1 index value for the present patient was 28%, suggesting the malignant nature of the tumor.

The designation of KMP has been applied to cases in which vascular lesions occur in association with profound thrombocytopenia and hypofibrogenemia with fibrin degradation products. It is important to rule out visceral lesions in the liver, spleen, and retroperitoneum, and hematologic malignancies such as leukemia and other malignancies that can cause a similar cutaneous presentation such as neuroblastoma [12]. The disseminated intravascular coagulation is caused by an activation of the clotting cascade and the formation of intravascular fibrin, and can results in microangiopathic hemolysis and occasionally red cell fragmentation seen on the peripheral blood smear [13]. Since the characteristic of tumor in this case was hypervascularity with bleeding and necrotic lesions, coagulopathy was thought to be caused by the trapping of platelets within a large vasculized tumor mass. Only a few reports described the coincidence of consumption coagulopathy and angiomatous tumor associated with KMP in the adulthood [7,15]. Moreover, although KMP is believed to be caused by clotting and fibrinolysis within giant hemangiomas and despite several cases of giant GIST of the stomach being reported, no case of concomitant gastric GIST and KMP has been reported thus far [8]. Because the predisposition to bleeding (observed in KMP) is the primary cause of death, aggressive treatment is required.

Imatinib mesylate, which is a competitive inhibitor of certain tyrosine kinases including intracellular kinases ABL and the BCR-ABL fusion proteins present in some leukemias, and PDGFRA, is the first effective drug for GIST [[16,17], 18]. The use of Gleevec as an adjuvant is currently being explored with on going randomized phase III trials.

## Conclusion

Since the characteristic of tumor in this case was hypervascularity with bleeding and necrotic lesions, coagulopathy was thought to be caused by the trapping of platelets within a large vasculized tumor mass.

## Competing interests

The author(s) declare that they have no competing interests.

## Authors' contributions

**TW **designed the study and participated in the writing process. **KS, TS, HK and TE **designed the study, carried out the data and picture acquisition as well as bibliographic research, drafted and revised the manuscript. **YJ, SK, NT, JS and TS **participated in manuscript revision process. **TS, TA, HN and TO **they participated in the editing process. All authors read and approved the final manuscript.
